# The Ontario Veterinary College, Toronto, Canada

**Published:** 1890-12

**Authors:** 


*The Ontario Veterinary College, Toronto, Canada.*—The opening lecture of this college was delivered by Dr. Duncan, Professor of Anatomy, on October 23d last. A large number of students were present, who come here, many from great distances, to pursue their professional studies, with the view of fitting themselves to successfully practice the science of veterinary medicine and surgery in after life. The gathering comprises representatives from all parts of Canada, even from British Columbia, the Pacific coast, and the far Northwest, as well as a large proportion from the United States. The Californian, the Westerner from Illinois and the prairies, the horseman of Kentuckey, and the Southerner from the Carolinas and Georgia, may each be found, studious in the lecture room and earnest in the dissecting room, with the one object in view. The new buildings which were occupied for the first time last January, that is, in the middle of last session, have proved to be, in suitability and convenience, all that its projector, Prof. Smith, could wish. The principal lecture hall is perfect in its acoustics, ventilation and warmth. The smaller rooms are equally comfortable. The building devoted to dissection is situated a short distance from the college buildings; it is large and commodious, and gives equal satisfaction. Dissection commenced a week or two before the opening lecture.

